# Looming sounds are perceived as faster than receding sounds

**DOI:** 10.1186/s41235-016-0017-4

**Published:** 2016-11-14

**Authors:** John G. Neuhoff

**Affiliations:** Department of Psychology, The College of Wooster, Wooster, OH 44691 USA

**Keywords:** Auditory looming, Auditory motion perception, Adaptation

## Abstract

Each year thousands of people are killed by looming motor vehicles. Throughout our evolutionary history looming objects have posed a threat to survival and perceptual systems have evolved unique solutions to confront these environmental challenges. Vision provides an accurate representation of time-to-contact with a looming object and usually allows us to interact successfully with the object if required. However, audition functions as a warning system and yields an anticipatory representation of arrival time, indicating that the object has arrived when it is still some distance away. The bias provides a temporal margin of safety that allows more time to initiate defensive actions. In two studies this bias was shown to influence the perception of the speed of looming and receding sound sources. Listeners heard looming and receding sound sources and judged how fast they were moving. Listeners perceived the speed of looming sounds as faster than that of equivalent receding sounds. Listeners also showed better discrimination of the speed of looming sounds than receding sounds. Finally, close sounds were perceived as faster than distant sounds. The results suggest a prioritization of the perception of the speed of looming and receding sounds that mirrors the level of threat posed by moving objects in the environment.

## Significance

Moving motor vehicles injure and kill hundreds of thousands of pedestrians each year. This situation has the potential to get dramatically worse as both the production of quieter electric and hybrid vehicles and the number of pedestrians distracted by hand-held electronic devices increase. However, a better understanding of the perceptual and cognitive aspects of looming sounds could lead to a reduction in these fatalities. These two experiments show that auditory perception of the speed of sounding objects in motion is related to their direction of travel and their distance from the listener. Sound sources that pose the greatest risk, those that are close and those that approach, are perceived to move faster than those that are distant or moving away. This perceptual bias suggests an evolutionary influence on our perception of looming auditory motion.

## Background

In the five-year period from 2009 to 2013, over 20,000 pedestrians were killed in the United States by moving motor vehicles (National Highway Traffic Safety Administration, 2015). Throughout our evolutionary history, looming objects have been a similar source of potential danger and perceptual systems have evolved in advantageous (though not perfect) ways to deal with such threats. The visual system provides a relatively accurate and precise estimate of arrival time that allows us to interact with looming objects effectively (McBeath, Shaffer, & Kaiser, 1995; Regan & Vincent, 1995). However, our field of vision is limited and of little value under poor viewing conditions (e.g. night-time) or when approaching objects are occluded. Audition is particularly useful under these conditions and typically functions as a warning system that gives an added measure of safety in dealing with looming objects (Guski, 1992; Rosenblum, Carello, & Pastore, 1987; Rosenblum, Wuestefeld, & Saldana, 1993). Looming sounds initiate a series of protective physiological, cognitive, emotional, and behavioral responses that do not occur in response to sounding objects that move in any other direction. Compared to equivalent receding sounds, looming sounds preferentially activate the amygdala and a distributed neural network that supports attention, auditory space and motion perception, and motor planning—all responses indicative of an adaptive trait that has evolved to keep organisms safe (Bach et al., 2008; Bach, Furl, Barnes, & Dolan, 2015; Bach, Neuhoff, Perrig, & Seifritz, 2009; Seifritz et al., 2002).

When listeners are asked to predict the arrival time of a looming sound, they exhibit a systematic anticipatory error and perceive that the source has arrived when it is still some distance away^1^ (Neuhoff, Hamilton, Gittleson, & Mejia, 2014; Neuhoff, Long, & Worthington, 2012; Neuhoff, Planisek, & Seifritz, 2009; Riskind, Kleiman, Seifritz, & Neuhoff, 2014; Rosenblum et al., 1987, 1993; Schiff & Oldak, 1990). This bias can provide a selective advantage by creating a temporal “margin of safety” that affords slightly more time than expected to initiate defensive behaviors in response to the looming object (Freiberg, Tually, & Crassini, 2001; Glatz, Bulthoff, & Chuang, 2014; Neuhoff, 1998, 1999, 2001). Studies on sex differences have shown that women tend to exhibit a larger looming bias than men (Grassi, 2010; Schiff & Oldak, 1990) and these findings are likely due to sex differences in the ability to deal with approaching threat (e.g. physical strength) rather than any differences in auditory spatial localization. For example, Neuhoff et al. (2009) showed that women perceive looming sounds as closer than men do. However, there was no difference between men and women in the perception of sounds that traveled away from the listener. Moreover, the magnitude of the looming bias is negatively correlated with physical strength and cardiac fitness (Neuhoff et al., 2012), consistent with the interpretation that the ability to deal effectively with a looming threat contributes to the magnitude of the bias. Individuals least prepared physically to deal with the oncoming threat have the largest perceptual margin of safety. Additional converging evidence for the adaptive looming bias hypothesis comes from comparative work on rhesus monkeys who show a pattern of response to looming versus receding sounds that mirrors that found in humans (Ghazanfar, Neuhoff, & Logothetis, 2002; Maier, Chandrasekaran, & Ghazanfar, 2008; Maier & Ghazanfar, 2007; Maier, Neuhoff, Logothetis, & Ghazanfar, 2004).

The auditory looming bias is part of a family of cognitive and perceptual biases that fall under the umbrella of “Error Management Theory” (Haselton et al., 2009; Haselton & Buss, 2000; Haselton & Nettle, 2006). Error Management Theory proposes that cognitive biases will evolve when judgments are made under conditions of uncertainty, when the decisions have historically had an impact on evolutionary fitness, and when there is an asymmetric cost of making false-positive and false-negative errors. There is a degree of uncertainty in any perceptual judgment and errors in judging auditory arrival time can clearly impact evolutionary fitness. The false-positive of responding too early is far less costly than the false-negative of responding too late.

From a psychophysical perspective, previous work has debated whether auditory motion is perceived directly or simply inferred from changes in distance by using “snapshots” at successive locations, with the bulk of the evidence suggesting that the auditory system can do both depending on the conditions (Carlile & Leung, 2016; Grantham, 1997). Egocentric auditory distance perception has already been shown to be a contributing factor in the auditory looming bias. For example, when listeners are asked to make stationary distance judgments of looming and receding sounds that have come to stop at the same egocentric distance from the listener, looming sounds are perceived as closer than receding sounds (Neuhoff, 2001; Neuhoff et al., 2009). However, there may also be a bias in speed perception. Differential estimates of speed for looming versus receding sounds could provide additional support for Error Management Theory and be consistent with an evolutionary perspective on the role of adaptive processes in auditory perception.

From a neurophysiological perspective, auditory receptive fields have been shown to be dynamic in nature and shift toward the motion of the sound, with the magnitude of these receptive field shifts increasing as the speed of the sound source increases (Witten, Bergan, & Knudsen, 2006). Although this work examined sounds that moved in azimuth (around the head), there may be similar mechanisms at work in processing looming sounds. Studies with humans and other animals have identified neural correlates of looming sounds and have implicated both cortical and subcortical regions that respond preferentially to looming sounds (Bach et al., 2008, 2009; Cappe, Thelen, Romei, Thut, & Murray, 2012; Ghazanfar et al., 2002; Maier & Ghazanfar, 2007; Seifritz et al., 2002). Similar work with auditory motion in azimuth has shown that a sounding object that enters an auditory receptive field produces more robust responding than one leaving it (Ingham, Hart, & McAlpine, 2001; Wilson & O’Neill, 1998). Thus the differential effects of increasing stimulus speed could be enhanced for looming sounds over receding much like looming and receding sounds of the same speed produce differential neural responses.

If the looming bias is an adaptive trait that has evolved as a protection mechanism against approaching danger, then we might expect listeners to perceive the speed of looming sounds as faster than that of equivalent receding sounds. We might also expect listeners to show better discrimination for the speed of looming sounds than for the speed of receding sounds because approaching sounds are more salient. Finally, we might expect listeners to perceive close sounds as faster than distant sounds because of their greater potential for danger. In two experiments we tested these hypotheses by presenting listeners with looming and receding sound sources and asking them to judge how fast they were moving.

## Experiment 1

### Method

#### Participants

The sample for Experiment 1 consisted of 80 participants (26 women) with an average age of 33.5 years (SD 9.9). Sample size was determined by examining prior auditory looming studies that used terminal distance as the dependent variable (rather than the speed estimates used here). These studies have typically used sample sizes of around 40 (e.g. Neuhoff et al., 2009). However, this sample size was doubled because of greater potential variability in the current experiment due to the online nature of the data collection (participants listened to the stimuli and responded on their own equipment). Data were collected until the target sample of 80 was reached. All participants reported normal hearing. All were recruited via Amazon Mechanical Turk (MTurk) and were paid $0.30 to complete the experiment online. A wide variety of research shows that samples from MTurk have reliability that is as good as or better than that obtained from traditional undergraduate samples (Buhrmester, Kwang, & Gosling, 2011; Holden, Dennie, & Hicks, 2013; Paolacci, Chandler, & Ipeirotis, 2010). Moreover, traditional attention and psychophysical tasks have been well replicated on MTurk including experiments on the Stroop effect, flanker task, Simon effect, Attentional Blink, task switching, inhibition of return, and masked priming (Crump, McDonnell, & Gureckis, 2013). Three participants were eliminated from the analysis of Experiment 1 because they misunderstood the task and gave estimates of the “speed of sound” that were all more than 600 mph. The study was approved by the Institutional Review Board of The College of Wooster and all participants provided informed consent.

### Stimuli

**Fig. 1:**
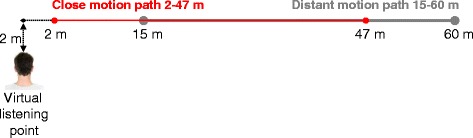
Stimulus configuration for Experiments 1 and 2. A virtual sound source approached or receded along a path parallel to the listener’s interaural axis at three different speeds. The listener was situated 2 m from the straight-line path of the source facing perpendicular to the path. In the “Close” condition, the sound source traveled between 2 m and 47 m from the median plane of the listener. In the “Distant” condition, the source traveled between 15 m and 60 m from the median plane of the listener

Stimuli consisted of a moving three-dimensional (3D) virtual sound source presented over headphones that traveled on a path parallel to the interaural axis of the listener. The virtual listening point was situated 2 m from the straight-line trajectory of the source (see Fig. 1). “Distant” stimuli approached and receded along a path between 60 m and 15 m from the median plane of the listener. “Close” stimuli approached and receded along a path between 2 m and 47 m from the median plane of the listener. Stimuli traveled at 15 mps (33.5 mph), 20 mps (44.7 mph), and 25 mps (55.9 mph). The sound source was a square wave with a fundamental frequency of 400 Hz and a sampling rate of 44.1 kHz. The virtual source height was 0.5 m. The simulation produced realistic 3D auditory motion that included Doppler shift, atmospheric filtering, gain attenuation due to atmospheric spreading, ground reflection attenuation, and head-related transfer function (HRTF) from the MIT KEMAR dataset (Gardner & Martin, 1995; see Neuhoff et al., 2009 for simulation details). We used a bypass trajectory to maximize interaural cues to the source’s approach. Stimuli are available at http://www.jneuhoff.com/links.html.

### Design and procedure

After providing informed consent, participants were asked by a recorded voice to adjust their volume to a comfortable listening level. As verification that they could hear the stimuli, they were asked to enter a code word into a blank text box, then to indicate the model headphones that they were using. They then chose the unit of speed that they were most familiar with (mph or km/h) to make speed estimates. All estimates in were later transformed to mps for analysis. Participants were instructed to listen to the audio clip and then estimate to the best of their ability how fast the sound source was traveling. Each participant heard two sounds in each condition (2 Distance × 2 Direction × 3 Speed) for a total of 24 trials. One sound in each condition approached/receded from the left. The other approached/receded from the right. The two responses in each condition were averaged to yield a single speed estimate for each condition. After hearing each sound, participants typed their speed estimate into a blank text box and clicked an arrow on the screen to advance to the next sound. Previous work has used a similar labeling method for assessing perceived speed (Recarte, Conchillo, & Nunes, 2000; Recarte & Nunes, 1996; Triggs & Berenyi, 1982).

## Results and discussion

**Fig. 2:**
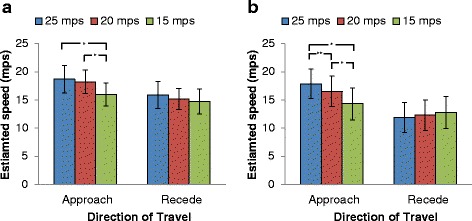
Mean speed estimates for each speed and direction condition collapsed across the two distances. The interaction of stimulus speed and direction of travel was significant in (**a**) Experiment 1, within-subjects design (n = 77) and (**b**) between-subjects design (n = 200). In both studies listeners show significant speed discrimination for looming sounds but no difference in the perceived speed of receding sounds. Error bars represent 95 % confidence intervals. * *p* < 0.01, ** *p* < 0.05

Participants generally underestimated actual speed (except in the slowest condition). However, this is a typical finding when participants are asked to verbally estimate speed (Recarte & Nunes, 1996; Triggs & Berenyi, 1982). Thus, our analysis is concerned only with relative differences between conditions, as accuracy in verbally applying metric values to perceptual stimuli typically shows much poorer performance than a motor response (Andre & Rogers, 2006), particularly with looming sounds (Neuhoff, 2001). A 2 (Direction) × 2 (Distance) × 3 (Speed) Repeated-Measures Analysis of Variance (ANOVA) revealed a main effect for Direction indicating that looming sounds (M = 39.1, SE = 2.3) were perceived as moving significantly faster than receding sounds (M = 32.2, SE = 2.3), F (1,76) = 17.8, *p* < 0.001, η_*p*_^*2*^ = 0.19. There was also a much larger main effect for Distance indicating that close sounds (M = 40.6, SE = 2.3) were perceived as moving faster than distant sounds (M = 30.8, SE = 2.1), F (1,76) = 76.6, *p* < 0.001, η_*p*_^*2*^ = 0.50, and a smaller main effect for Speed (see Table 1), F (1.83,76) = 5.9, *p* = 0.005, η_*p*_^*2*^ = 0.07 (Greenhouse-Geisser correction used to correct for sphericity violation). However, this effect was moderated by a significant interaction between Direction and Speed, F (2,152) = 4.6, *p* = 0.01, η_*p*_^*2*^ = 0.06. Scores were collapsed across Distance to examine this interaction and separate ANOVAs were performed on the Looming and Receding trials with Speed as the only independent variable. The analysis revealed a main effect of Speed for looming sounds F (2,68) = 3.1, *p* = 0.05, η_*p*_^*2*^ = 0.08, indicating that listeners could discriminate among the different speeds of looming sounds (See Fig. 2a). Post-hoc Tukey tests revealed significant differences in perceived speed between the 25 mps and 15 mps conditions and between the 20 mps and 15 mps conditions, *p* < 0.05. Importantly, there was no significant difference among the perceived speeds of receding sounds, F (2,68) = 0.81, *p* = 0.49, η_*p*_^*2*^ = 0.02. Finally, there was a significant interaction of Distance and Direction, F (1,76) = 13.3, *p* < 0.001, η_*p*_^*2*^ = 0.15. Follow-up analyses showed a significant difference in perceived speed between close looming sounds (M = 45.9, SD = 24.1) and close receding sounds (M = 35.2, SD = 21.6), t(76) = 4.7, *p* < 0.001. There was also a significant difference between distant looming sounds (M = 32.4, SD = 18.6) and distant receding sounds (M = 29.3, SD = 22.3), t(76) = 2.0, *p* = 0.04. However, the effect size for the difference between close looming and receding sounds (d = 0.47) was over three times the effect size for the difference between distant looming and receding sounds (d = 0.15), indicating a perceptual priority for speed discrimination among sounds that are close. The three-way interaction of Direction × Distance × Speed was not significant F (2,152) = 1.28, *p* = 0.30, η_*p*_^*2*^ = 0.03.

**Table 1:** Mean estimates of perceived speed by condition in Experiments 1 and 2

	Experiment 1	Experiment 2
	M (SE)	M (SE)
15 mps	15.1 (2.4)	13.5(0.94)
20 mps	16.1 (1.0)	14.4(0.98)
25 mps	16.7 (0.9)	14.9(0.98)

Notes: The small significant main effects for speed in each experiment were moderated by significant interactions. Participants made speed estimates in either mph or km/h. All estimates were transformed to mps for analysis. 15 mps = 33.6 mph, 20 mps = 44.7 mph, 25 mps = 55.9 mph.

Looming sounds and close sounds were both perceived to move faster than equivalent receding and distant sounds. These results indicate a perceptual priority for sounds most critical to the listener. In a natural environment sounds that are close and sounds that are approaching have the potential to pose the greatest danger. The interaction between Speed and Direction supports this position by showing that listeners have greater sensitivity to the speed of auditory motion when a sound source is approaching than when it is receding. The significant interaction between Distance and Direction is also consistent with the proposal.

## Experiment 2

Experiment 1 showed that looming sounds and close sounds are perceived to move faster than equivalent receding and distant sounds and that listeners show preferential perceptual processing for sounds that have the most potential for danger. However, it is possible that the within-subjects nature of the experimental design tipped participants off to the hypothesis and created demand characteristics for our evolutionary hypotheses about direction and distance. In Experiment 2, we used the same stimuli and procedure but made Distance and Direction between-subjects factors. Thus, each participant provided speed estimates for only three sounds (at 15 mps, 20 mps, 25 mps) in one of the four conditions (close-looming, close-receding, distant looming, or distant-receding). This design eliminated any potential demand characteristics on our primary variables of interest.

### Method

#### Participants

The sample for Experiment 2 consisted of 200 participants (77 women) with an average age of 32.1 years (SD 10.1). Sample size was determined by examining prior auditory looming studies that used between-subjects variables. These studies have typically used sample sizes of around 30 per condition (Neuhoff et al., 2014, 2009). However, this sample size was increased because of greater expected variability in the current experiment due to the online nature of the data collection. Data were collected until the target sample of 50 per each of the four conditions was reached. All participants reported normal hearing. All were recruited via MTurk and were paid $0.30 to complete the experiment online. Eleven participants were replaced because they misunderstood the task and gave estimates of the “speed of sound” that were over 600 mph. The study was approved by the Institutional Review Board of The College of Wooster and all participants provided informed consent.

### Stimuli

The stimuli were the same as those used in Experiment 1.

### Design and procedure

The procedure was the same as that used in Experiment 1. However, in the between-subjects design of Experiment 2, each participant heard three sounds (one at each velocity) in only one of the four (2 Distance × 2 Direction) conditions for a total of three trials. Half the participants heard the sounds approach/recede from the left. The other half heard sounds approach/recede from the right.

## Results and discussion

A 2 × 2 × 3 Mixed-Design ANOVA was conducted on the speed estimates with the between-subjects factors “Direction” (looming/receding) and “Distance” (2 m, 15 m) and the within-subjects factor “Speed” (15 mps, 20 mps, 25 mps). Looming sounds (M = 36.3, SE = 2.9) were again perceived as significantly faster than receding sounds (M = 27.6, SE = 2.9), F(1, 196) = 4.4, *p* < 0.037, η_*p*_^*2*^ = 0.02. Close sounds (M = 36.9, SE = 2.9) were again perceived as faster than distant sounds (M = 27.0, SE = 2.9), F(1, 196) = 5.7, *p* = 0.018, η_*p*_^*2*^ = 0.03. There was also a significant effect for Speed (see Table 1), F(1.9, 392) = 3.2, *p* = 0.045, η_*p*_^*2*^ = 0.02 (Greenhouse-Geisser correction used to correct for sphericity violation). The main effect for Speed was once again moderated by a significant Speed × Direction interaction, F(2, 392) = 9.1, *p* < 0.001, η_*p*_^*2*^ = 0.05. Separate follow-up ANOVAs for Looming and Receding conditions with Speed as the within-subjects variable indicated a main effect for Speed of looming sounds, F(2, 198) = 18.2, *p* < 0.001, η_*p*_^*2*^ = 0.16, indicating that listeners could discriminate among the different speeds of looming sounds (See Fig. 2b). Post-hoc Tukey tests showed significant differences between all three speed conditions for looming sounds, *p* < 0.05. However, there was no significant difference in the speed estimates of receding sounds F(2, 198) = 0.07, *p* = 0.93, η_*p*_^*2*^ < 0.001 The interactions between Distance × Direction F(2, 198) = 0.05, *p* = 0.82, η_*p*_^*2*^ < 0.01, Speed × Distance F(2, 392) = 0.07, *p* = 0.82, η_*p*_^*2*^ < 0.01, and the three-way interaction of Direction × Distance × Speed F(2, 198) = 0.05, *p* = 0.82, η_*p*_^*2*^ < 0.01 were not significant.

The results of Experiment 2 replicate the main findings of Experiment 1. Listeners treat looming sounds and close sounds with perceptual priority. They perceive them as moving faster than receding and distant sounds of equal speed. Listeners also have greater speed discrimination for looming sounds than for receding sounds even when they only hear sounds that travel in one direction.

### General discussion

The current results show that the auditory system prioritizes the perceptual processing of looming sounds in a way that mirrors their potential for danger based on their proximity to the listener and their direction of travel. Close looming sounds pose the greatest potential threat and are perceived as moving faster than equivalent receding sounds. Listeners also show greater speed discrimination for looming sounds than they do for receding sounds. These findings are consistent with the interpretation that the perception of auditory motion has been shaped in part by the environmental challenges posed by our ancestors and that the auditory looming bias adaptation is specific to the conditions that pose the greatest potential for danger.

The results also show a bias in speed perception of looming sounds that occurs in the absence of distance-related tasks. Although listeners may sometimes use changes in distance to estimate speed, there is also good evidence for the direct detection of auditory speed (Carlile & Best, 2002; Freeman et al., 2014; Locke, Leung, & Carlile, 2016; Warren, Zielinski, Green, Rauschecker, & Griffiths, 2002). It is notable that speed estimates in all but the slowest stimulus condition were generally less than the actual speed of the stimuli. Recent psychophysical work has also shown an underestimation of auditory speed in a circular pattern around the head (Senna, Parise, & Ernst, 2015). However, the current finding is likely the result of a well-documented difficulty in applying metric labels accurately to speed stimuli rather than a true perceptual underestimation of the speed of approach (Recarte et al., 2000; Recarte & Nunes, 1996; Triggs & Berenyi, 1982). For example, previous research using the same stimuli required participants to execute a button push when a looming sound source arrived and participants significantly underestimated arrival time indicating a perceived speed that was faster than actual (Neuhoff et al., 2014, 2012; Riskind et al., 2014). Because “labeling” the speed of looming sounds is typically not a priority in a natural environment, the primary concern here was to examine relative differences between conditions rather than absolute estimates of speed. Moreover, participants show better accuracy with a motor response than a verbal response in judging looming stimuli (Neuhoff, 2001).

The auditory looming bias is technically a systematic error in our perception of looming sounds. However, Error Management Theory proposes that a wide range of social, cognitive, and perceptual biases have evolved because they increase the likelihood of survival and reproduction (Haselton et al., 2009; Haselton & Buss, 2000; Haselton & Nettle, 2006). Perceptual “errors” can provide a selective advantage if they offer survival benefits that exceed those obtained from veridical perception. The differential benefit of the looming bias is demonstrated if we contrast a listener with veridical perception to a listener with a bias to hear looming sounds as closer than they are. On average, the listener with veridical perception perfectly predicts the arrival time of the source and the listener with the looming bias responds consistently early. However, each listener has also a degree of variability associated with their arrival time judgments, each sometimes responding slightly earlier or slightly later than their respective means. Early judgments that provide more time than expected to prepare for the arrival of the source are not problematic for either listener. However, a listener with veridical perception who responds just a half second late is responding after the source has already arrived, whereas late response by the listener with the looming bias may still leave enough time to respond safely.

It is likely that the looming bias is an automatic process not under conscious control. Several studies have shown increased autonomic nervous system responses to looming sounds consistent with a protective mechanism (Bach et al., 2009; Fletcher et al., 2015; Tajadura-Jimenez, Valjamae, Asutay, & Vastfjall, 2010). If the decision to engage the motor system in the face of a looming sound source was entirely under conscious control, then anyone engaged with a high cognitive load at the time would be disadvantaged in that there would be fewer cognitive resources to devote to the approaching danger. However, a recent study that manipulated cognitive load while participants judged the arrival of a looming sound found just the opposite. McGuire, Gillath, and Vitevitch (2016) asked listeners to judge when a looming sound would reach them while under high cognitive load (memorizing a seven-digit number) or low cognitive load (memorizing a two-digit number). They found that the looming bias was significantly larger under high cognitive load. The finding that listeners respond sooner rather than later under high cognitive load suggests that the bias to hear sounds as closer than actual is an automatic process that requires little effortful cognitive processing. However, conscious attention can in some cases influence time-to-arrival estimates as some studies have shown that repeated trials with feedback can reduce (but not eliminate) the auditory looming bias (Rosenblum, Gordon, & Wuestefeld, 2000).

The bias to hear looming sounds as closer and faster than equivalent receding sounds is not well predicted by any traditional psychophysical laws. Some of the bias as tested in loudness change experiments may be due to an “endpoint bias” (Canevet, Teghtsoonian, & Teghtsoonian, 2003; Teghtsoonian, Teghtsoonian, & Canevet, 2005), where the end loudness of a rising intensity sound that changes from 60 to 75 dB has a greater influence on loudness change estimates than the end loudness of a falling intensity sound that changes from 75 to 60 dB. The argument is that with equal endpoints in loudness the bias might disappear. However, when listeners are asked to make terminal distance estimates of real looming and receding sounds in a real or virtual environments, looming sounds are perceived as significantly closer despite the same distance (or endpoint) from the listener (Neuhoff, 2001; Neuhoff et al., 2009). Thus, with real looming sounds, the endpoint bias disappears. More importantly, any “psychophysical explanation” for the effect would not imply that the looming bias is not an evolutionary adaptation. Psychophysics does not preclude evolution and if there were a good psychophysical description of the effect, it is highly likely that the causal direction would go from the evolved adaptation to the psychophysical result (Cauchoix & Chaine, 2016; Croston, Branch, Kozlovsky, Dukas, & Pravosudov, 2015; Warren, 1981).

## Conclusions

The current results show that moving sounds are perceived as traveling fastest when they are in a position to pose the greatest threat. They provide evidence for an evolved perceptual auditory looming bias. Further support for the auditory looming bias as an adaptation comes from several converging lines of research. Neuroimaging studies have identified specific neural mechanisms that preferentially process looming sounds over auditory motion in other directions (Bach et al., 2008, 2009, 2015; Seifritz et al., 2002). Sex differences and a correlation between physical fitness and the looming bias suggest that the bias is related to the ability to defend oneself in the face of a looming threat (Grassi, 2010; Neuhoff et al., 2009, 2012, 2014; Schiff & Oldak, 1990). Behavioral experiments show that listeners consistently err on the side of safety when perceiving auditory arrival time (Gordon & Rosenblum, 2005; Neuhoff, 2001; Rosenblum et al., 1987, 1993, 2000). Developmental work shows that the bias is present at a very early age (Freiberg et al., 2001; Morrongiello, Hewitt, & Gotowiec, 1991) and comparative research shows that the bias occurs in a species closely related to humans (Ghazanfar et al., 2002; Ghazanfar & Maier, 2009; Maier et al., 2004; Maier & Ghazanfar, 2007). Together these results provide strong support for an evolved bias in the perception of looming sounds.

Though our everyday environment has been dramatically and rapidly changed by industry and technology. Many of the evolved cognitive and perceptual adaptations that we inherited from our ancestors remain. A greater understanding of these evolutionary adaptations can facilitate solving some of our modern day problems.

## Footnotes

1 There is some evidence that suggests that visual time-to-arrival is also underestimated, though not nearly as much as in audition (e.g. Schiff & Oldak 1990). However, this work employs a methodology that occludes that last portion of approach of the looming object. Thus, the task is really a prediction of the arrival time of an unseen object based on a previously seen trajectory. Under unoccluded conditions, humans show very high accuracy in judging visual arrival time and a systematic anticipatory bias in judging auditory arrival time. When a looming object (auditory or visual) suddenly disappears on approach, there is simply no stimulus to perceive. Thus, the task changes from one of “perception” to one of predictive “decision making.” It is likely that when one can no longer hear nor see a previously looming object, there is a common tendency to make conservative judgments in both modalities. However, this process is not “perception” per se. Nonetheless, there are some visual studies that find asymmetries in favor of looming visual motion. For example, observers have longer motion aftereffects for looming versus receding visual stimuli and perceive ambiguous egocentric motion more often as looming than receding (“Lewis and McBeath, 2004”; Scott, Lavender, McWhirt, & Powell, 1966). On the other hand, some work has shown a bias toward receding visual motion (Edwards & Ibbotson, 2007; Mueller & Timney, 2014). As such, it is still an open question as to whether the perception of looming objects represents “amodal perception” across the auditory and visual modalities (Gordon & Rosenblum 2005; Rosenblum 2008)
